# In–Situ Cartilage Functionality Assessment Based on Advanced MRI Techniques and Precise Compartmental Knee Joint Loading through Varus and Valgus Stress

**DOI:** 10.3390/diagnostics11081476

**Published:** 2021-08-14

**Authors:** Oliver Said, Justus Schock, Daniel Benjamin Abrar, Philipp Schad, Christiane Kuhl, Teresa Nolte, Matthias Knobe, Andreas Prescher, Daniel Truhn, Sven Nebelung

**Affiliations:** 1 Department of Diagnostic and Interventional Radiology, Aachen University Hospital, 52074 Aachen, Germany; osaid@ukaachen.de (O.S.); pschad@ukaachen.de (P.S.); ckuhl@ukaachen.de (C.K.); tnolte@ukaachen.de (T.N.); dtruhn@ukaachen.de (D.T.); snebelung@ukaachen.de (S.N.); 2 Department of Diagnostic and Interventional Radiology, Medical Faculty, University Dusseldorf, 40225 Dusseldorf, Germany; DanielBenjamin.Abrar@med.uni-duesseldorf.de; 3 Department of Orthopedic and Trauma Surgery, Lucerne Cantonal Hospital, 6000, Lucerne, Switzerland; matthias.knobe@luks.ch; 4 Institute of Molecular and Cellular Anatomy, RWTH Aachen University, 52074 Aachen, Germany; aprescher@ukaachen.de

**Keywords:** loading, stress MRI, varus, valgus, knee joint, cartilage

## Abstract

Stress MRI brings together mechanical loading and MRI in the functional assessment of cartilage and meniscus, yet lacks basic scientific validation. This study assessed the response-to-loading patterns of cartilage and meniscus incurred by standardized compartmental varus and valgus loading of the human knee joint. Eight human cadaveric knee joints underwent imaging by morphologic (i.e., proton density-weighted fat-saturated and 3D water-selective) and quantitative (i.e., T1ρ and T2 mapping) sequences, both unloaded and loaded to 73.5 N, 147.1 N, and 220.6 N of compartmental pressurization. After manual segmentation of cartilage and meniscus, morphometric measures and T2 and T1ρ relaxation times were quantified. CT-based analysis of joint alignment and histologic and biomechanical tissue measures served as references. Under loading, we observed significant decreases in cartilage thickness (*p* < 0.001 (repeated measures ANOVA)) and T1ρ relaxation times (*p* = 0.001; medial meniscus, lateral tibia; (Friedman test)), significant increases in T2 relaxation times (*p* ≤ 0.004; medial femur, lateral tibia; (Friedman test)), and adaptive joint motion. In conclusion, varus and valgus stress MRI induces meaningful changes in cartilage and meniscus secondary to compartmental loading that may be assessed by cartilage morphometric measures as well as T2 and T1ρ mapping as imaging surrogates of tissue functionality.

## 1. Introduction

Magnetic Resonance Imaging (MRI) is considered the most powerful and multifaceted imaging technique of modern medicine and provides the reference standard for joint assessment. Even though clinical MRI techniques are characterized by good-to-excellent diagnostic accuracy in the evaluation of the knee joint [1], early degenerative changes of articular cartilage and the medial and lateral menisci remain difficult to diagnose based on clinical standard MRI techniques [2,3]. Detecting early cartilage and meniscus degeneration at the pre-structural level is merited by the fact that early degeneration may still be reversible. Once structural damage of cartilage and meniscus becomes visible, the damage to the tissue matrix is likely irreversible [4].

Despite recent advances in knee MRI acquisition and processing that relate to morphologic and compositional cartilage imaging, automated image analysis, and hardware improvements (excellently reviewed in [5]), the unphysiological patient position in the MRI scanner, i.e., supine with the joints completely unloaded, may be partially responsible for the diagnostic shortcomings of clinical standard MRI techniques [6,7]. Consequently, recent approaches have combined MRI techniques with simultaneous loading to assess the tissue’s response to loading as an imaging surrogate parameter of its functionality. Recently, static mechanical loading has been combined with continuous 3D-MRI acquisition to measure the intra-tissue strain of articular cartilage [8], while earlier approaches aimed to determine displacement under applied loading by synchronizing loading with the MRI acquisition [9]. Principally, imaging of the knee joint under loading is possible in open low-field MRI scanners (i.e., B_0_ ≤ 0.5 T) where the patient bears weight [10] or in closed-bore high-field (clinical) MRI scanners (i.e., B_0_ ≥ 1.5 T) where the patient is loaded by prototypical devices. These devices are positioned in the horizontal bore alongside the patient and make use of optimized signal-to-noise ratio, image resolution, and examination times afforded by higher magnetic field strengths. Such devices most often apply axial loading along the lower extremity’s mechanical axis [7]. However, their handling is oftentimes inconvenient in scientific (and clinical) practice as these devices use suspended weights and pulley systems [11] or induce indirect compressive loading by control of displacement [12] or pressure [13]. Additionally, the lower extremity must be mechanically immobilized as the joint necessarily undergoes flexion and tibial rotation [14], thereby challenging intra- and inter-patient reproducibility and standardization [11,13].

To overcome these difficulties, our group recently proposed an alternative loading mechanism that applies compartmental pressurization of the medial or lateral femorotibial compartment by varus or valgus loading along the joint line, i.e., perpendicular to the mechanical axis [15]. Preliminary evidence in a single human cadaveric knee joint indicated efficient areal pressurization with average decreases in cartilage thickness of 6–9% (medial compartment) and 3–7% (lateral compartment) in response to loading of 15 kp.

The present study aimed to further substantiate these preliminary findings by (i) studying larger sample sizes, by (ii) using quantitative MRI techniques, i.e., T2 and T1ρ mapping, to evaluate the effects of loading on the compositional and (ultra-)structural levels, and by (iii) referencing the loading-induced changes to histologic and biomechanical tissue measures. Our hypotheses were that (i) precise varus and valgus stress MRI induces consistent compartmental pressurization and decreases in cartilage thickness of the loaded compartment (i.e., medial under varus loading and lateral under valgus loading), that (ii) the loading-induced changes in tissue thickness, composition, and (ultra)structure are reflected by associated changes in the T2 and T1ρ maps, and that (iii) these changes are related to histologic and biomechanical measures of cartilage and meniscus.

## 2. Materials and Methods

### 2.1. Study Design and Sample Size Estimation

Designed as a prospective in-situ imaging study on human knee joint specimens with intra-individual histologic and biomechanical referencing, the present study had been approved by the responsible Institutional Review Board (Ethical Committee, RWTH Aachen University, AZ-EK180/16) prior to its initiation.

Eight fresh and structural intact knee joints (2 right, 6 left) were obtained from body donors (aged 79.3 ± 4.0 years (mean ± standard deviation)), who had deceased because of unrelated medical conditions. Consequently, body donors whose medical history indicated the presence of bone or knee joint pathology (such as established diagnoses of osteoarthritis, rheumatoid arthritis, soft tissue injuries, Paget’s disease, and others) were excluded a priori. The body donors’ written informed consent was available at study initiation. All relevant local guidelines and regulations were strictly obeyed. Minimum sample size was estimated as five by performing power analyses on the initial three knee joint specimens as is commonly performed in exploratory settings [16]. Using the following framework parameters: power 0.8; probability of type-I-error 0.05; two-tailed procedure; www.statstodo.com (accessed on 1 June 2018)), the effect size (defined as the mean of the paired difference to be detected divided by the expected standard deviation of the paired difference) was determined as 1.6 after measuring the T2 relaxation times of the unloaded and loaded configurations (to 15 kp varus) of a rectangular region-of-interest placed in the central weight-bearing cartilage of the medial femorotibial compartment. To reflect the anticipated variability between T2 and T1ρ, femur and tibia, medial and lateral, and the distinct varus and valgus configurations, the number of included specimens to be included was increased to eight.

### 2.2. MRI-Compatible Loading Device

**Figure A1:**
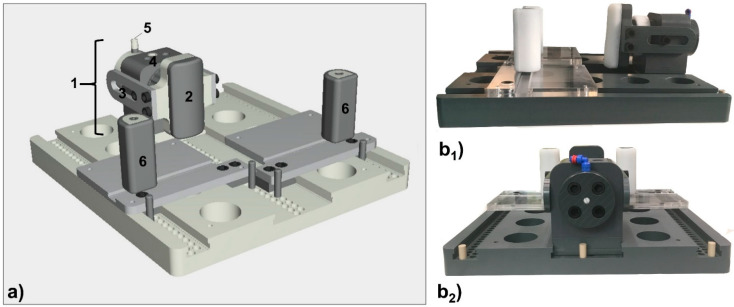
Loading unit of the MRI-compatible pressure-controlled loading device. (**a**) Schematic visualization of the components. (1) Pneumatic mechanism, (2) padded pressure applicator, (3) safety rails, (4) encasement, (5) pressure port, (6) padded counter-bearings. (**b**) Unloaded device as viewed from the side (**b_1_**) and the rear (**b_2_**).

The loading device for compartmental femorotibial compression has been validated before [15] and is shown in detail in Figure A1. Briefly, the device is controlled by pressure and consists of a control unit (outside of the scanner room) and a loading unit (inside the scanner room) that are connected by standard pressure lines. The device is designed along the leverage principle, where compartmental pressurization of the joint is realized by aligning the padded load applicator with the joint line. With two adjustable counter-bearings as opposite fixed points at the thigh and lower leg, the medial (or lateral) femorotibial compartments are loaded by medial (or lateral) pressurization of the joint by inducing varus (or valgus) stress. In the present study, forces of 7.5 kp (=73.5 N), 15 kp (=147.1 N), and 22.5 kp (=220.6 N) were applied to the joints to induce varus and valgus stress and, secondarily, medial and lateral compartment loading.

### 2.3. Preparation of The Knee Joint Specimens

For the MRI measurements, the knee joints were positioned in the loading device at full extension. Positioning aids and sandbags were used to fix the joints and standardize their positions, while reducing adaptive motion secondary to loading. Practically, the center of the padded load applicator was aligned with the medial and lateral joint line, while the counter-bearings were adjusted to the individual joint’s anatomy and positioned as distant from each other as possible at the lower third of the thigh and the upper third of the lower leg. For the unloaded reference measurements, the components were brought in loose contact with the joint. Subsequently, after connecting the control and loading units to the in-house pressure supply, the system was fully operational.

### 2.4. Imaging Studies

#### 2.4.1. MRI Studies

For scanning, a clinical 3.0T scanner (Achieva, Philips, Best, The Netherlands) and two-element general-purpose coils (Sense-Flex L, Philips) positioned above and below the joint were used. By means of B_0_ mapping, the absence of excessive magnetic field inhomogeneity had been determined before [15]. For each joint, serial MRI measurements were conducted in seven sequential configurations:

- unloaded (δ_0_);
- under low intensity of varus loading (7.5 kp, δ_var1_);
- under moderate intensity of varus loading (15 kp, δ_var2_);
- under high intensity of varus loading (22.5 kp, δ_var3_);
- under low intensity of valgus loading (7.5 kp, δ_vlg1_);
- under moderate intensity of valgus loading (15 kp, δ_vlg2_);
- and under high intensity of valgus loading (22.5 kp, δ_vlg3_).

After each change in pressure, an equilibration period of 5 min was observed prior to image acquisition. For each configuration, morphologic sequences, i.e., proton density-weighted fat-saturated (PD-fs) sequences and 3D water-selective cartilage scans (WATSc), and quantitative T2 and T1ρ mapping sequences were acquired as detailed in Table 1. We intended to include the clinical reference sequence for cartilage assessment, i.e., the PD-fs sequence [17], a representative and scientifically validated high-resolution sequence for cartilage segmentation and morphometric analyses, i.e., the WATSc sequence [18], and clinically validated quantitative T2 and T1ρ mapping sequences for assessment of tissue functionality [19]. Proper joint position at each successive configuration was confirmed using the PD-fs sequences. While the PD-fs sequences were acquired in three (δ_0_) or two orientations (δ_var1_ to δ_vlg3_), the WATSc and T2 and T1ρ mapping sequences were acquired in the coronal orientation only. For the T2 and T1ρ mapping sequences, only the mid-coronal images were analyzed to reduce the processing burden. Oriented parallel to the posterior condylar line, the mid-coronal plane was identified as the center of the anteroposterior distance between the posterior condylar line and the deepest point of the trochlear groove on axial views. MRI measurements were performed at room temperature and completed within 12 h.

During the unloaded initial scan, the femoral and tibial cartilage of the medial and lateral joint compartments was qualitatively assessed by SN (clinical radiologist, 8 years of experience in musculoskeletal imaging) and any specimens with areal full-thickness cartilage loss, denudation, or eburnation were discarded (two specimens).

#### 2.4.2. CT Studies

Following completion of the MRI studies, the knee joints underwent sequential scanning in the seven sequential configurations and based on the same loading device and configuration on a clinical multidetector-row CT scanner (SOMATOM Force, Siemens, Erlangen, Germany) using the following scan parameters: craniocaudal direction, tube voltage 120 kV, tube current 800 mAs, slice thickness 0.6 mm, rotation time 1 s, increment 3 mm, pitch 0.8, spatial resolution 0.31 × 0.31 mm/pixel, reconstruction kernel Br64s, scan duration 2 s (per configuration). Axial, coronal, and sagittal reconstructions of the joint in the device were generated for each configuration. Analogous to the MRI studies, equilibration periods of 5 min were observed after each change in pressure.

### 2.5. Image Post-Processing and Analyses

#### 2.5.1. MRI—Morphometric Analysis of Cartilage

For each joint and configuration, the 3D WATSc sequences were used for manual segmentations and morphometric analyses using dedicated software (Chondrometrics GmbH, Ainring, Germany) [20]. In each image, the cartilage surfaces and subchondral bone plates of the medial and lateral femorotibial compartments were labelled manually by blinded readers. An expert reader ascertained reading quality. Based on the segmentation outlines, 3D morphometric reconstructions of the cartilage plates were quantified in terms of cartilage thickness (ThC). Morphometric cartilage measures were thus obtained for the medial femorotibial compartment (MFTC), the lateral femorotibial compartment (LFTC), the medial tibia, the lateral tibia, the central medial femur, and the central lateral femur. For subregional analysis of cartilage changes under loading, the medial and lateral tibia were further partitioned into five subregions, i.e., central, external, internal, anterior, and posterior, while the central medial and lateral femur were further partitioned into three subregions, i.e., central, external, and internal.

#### 2.5.2. MRI—Quantitative Analysis

T2 and T1ρ characteristics were quantified for each cartilage plate, joint, and configuration. By manually delineating each structure using the polygon mode and brush tool of ITK-SNAP software (v3.8, Cognitica, Philadelphia, PA, US), OS (radiologist, 2 years of experience in musculoskeletal imaging) manually segmented the medial and lateral tibial and femoral cartilage plates and the medial (MM) and lateral meniscus (LM) on the respective mid-coronal images. Boundary pixels were eliminated to reduce partial volume effects. Segmentation outlines of the articular cartilage were automatically partitioned into three subregions, i.e., internal, central, and peripheral (from the joint center to the joint periphery), by using a dedicated routine implemented in MATLAB (MatlabR2019a, Natick, MA, US) that divided the horizontal dimension of the entire cartilage plate into thirds. Segmentation outlines were checked by SN and validated against the corresponding WATSc images. These post-processing routines were implemented in MATLAB as before [21,22].

For each joint and configuration, meniscal extrusion was measured by OS using the mid-coronal images of the PD-fs sequence and the in-house picture archiving and communication system (PACS, iSite^®^, Philips) and its standard image analysis features. To this end, the horizontal distance was determined between the tibia plateau’s point of transition from horizontal to vertical and the outermost contour of the medial or lateral meniscus body [23].

#### 2.5.3. CT

**Figure 1:**
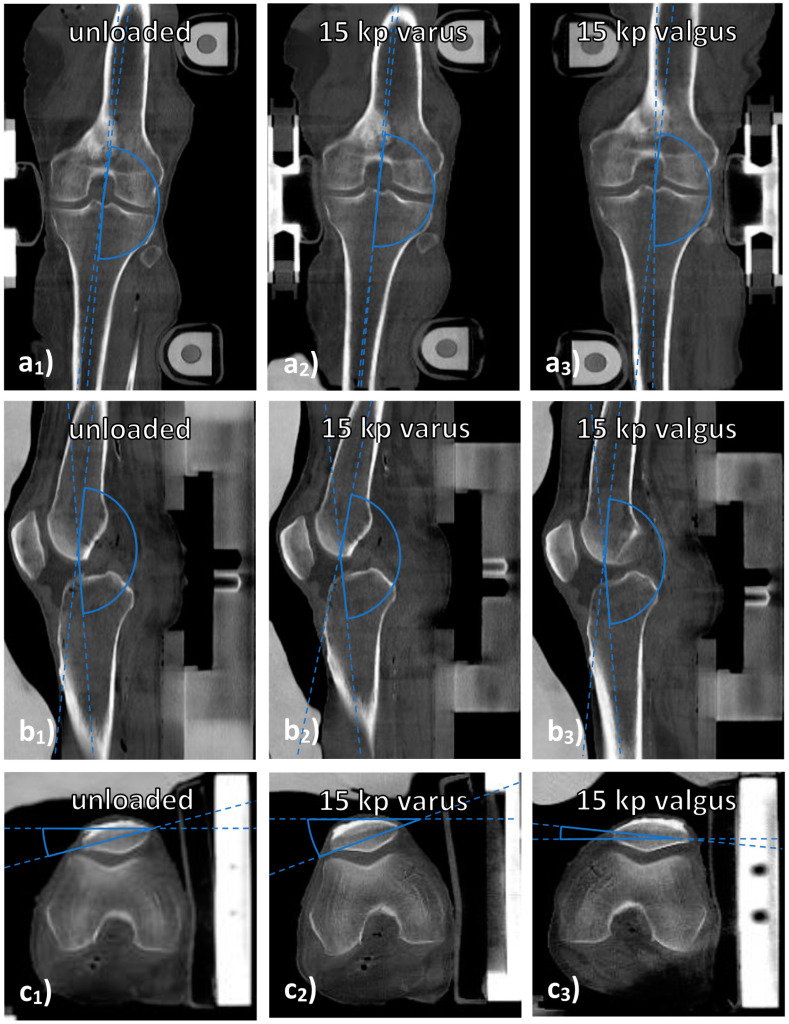
Measurements of joint alignment on CT images. Visualized are coronal (**a**), sagittal (**b**), and axial (**c**) reconstructions of a representative knee joint under various loading conditions, i.e., unloaded (**a_1_**–**c_1_**), under 15 kp varus loading (**a_2_**–**c_2_**) and under 15 kp valgus loading (**a_3_**–**a_3_**). “Joint deviation” was determined on mid-coronal images as the lateral angle between the femoral and tibial shafts (**a**). “Joint extension” was determined on mid-sagittal images as the popliteal angle between the femoral and tibial shafts (**b**). “Joint rotation” was determined on the axial images that demonstrated the largest diameter of the patella by relating the trans-patellar line (through the outer corners of the patella) to the horizontal line (**c**). By convention, external rotation was indicated by positive angles and internal rotation by negative angles.

CT datasets were analyzed to determine the joint’s alignment as a function of configuration. Because of the larger field of view, CT scans visualized the entire joint and loading device so that measures of joint alignment in the device could be taken on the respective CT scans. Joint alignment was quantified by determining the angles between the tibial and femoral shafts on sagittal (“joint extension” (popliteal angle)) and coronal reconstructions (“joint deviation” (lateral angle)) as well as between a line joining both corners of the patella and the horizontal line on axial reconstructions (“joint rotation”) (Figure 1). OS performed these measurements using the in-house PACS. Blinding proved impractical because each joint’s configuration was easily discernible.

### 2.6. Reference Measures

After imaging, the knee joints underwent histologic and biomechanical referencing. The joints were accessed through the medial parapatellar approach, and the cartilage surfaces and menisci were fully exposed after transection of the collateral and cruciate ligament complex. To align the mid-coronal plane (identified during imaging) with the central weightbearing portion of the femur and tibia (identified during post-hoc preparations), tissue-marking dye (Polysciences, Warrington, FL, US) was applied to mark the respective planes and to guide sampling of the adjacent joint surfaces. The medial and lateral meniscus body regions were sampled similarly.

#### 2.6.1. Histologic Reference 

Following simultaneous decalcification and fixation using Ossa fixona (Diagonal, Münster, Germany) for cartilage or fixation using 4% paraformaldehyde for meniscus, cartilage and meniscus were sectioned along the mid-coronal plane as identified above. Sectioned tissues were embedded in paraffin, sliced to 5-µm sections, stained with Safranin O and hematoxylin/eosin, and visualized using a standard light microscope (Leica DMI6000 B, Leica, Wetzlar, Germany) [24,25]. If necessary, individual micrographs were automatically stitched to obtain entire-sample visualizations by use of dedicated software applications (Leica Application Suite X, Leica). Semi-quantitative grading was individually performed by two investigators (OS and SN). Grading of the cartilage was based on a modified OARSI (Osteoarthritis Research Society International) grading system [26] that only considered the grade of degeneration (score, 0 (intact)—6 (most severe degeneration)) to describe the extent of degenerative changes along the cartilage thickness. Meniscus samples were graded in line with the Pauli classification [27] that takes into account surface integrity (score, 0–3 (for the femoral, tibial, and inner surface each)), cellularity (score, 0–3), collagen organization (score, 0–3), and matrix staining (score, 0–3). The itemized scores were summed (range, 0–18) and used to allocate the tissue to one of four grades, i.e., grade 1 (intact; sum score, 0–4), grade 2 (early degenerative; sum score 5–9), grade 3 (moderately degenerative; sum score, 10–14), and grade 4 (severely degenerative; sum score, 15–18). If scores differed between the readers, histologic sections were re-evaluated until consensus was reached.

#### 2.6.2. Biomechanical Reference

Cartilage-only tissue was prepared from the femoral and tibial surfaces by harvesting cartilage plugs of 8 mm diameter through a skin biopsy punch (pfm-medical, Cologne, Germany) and by removing the subchondral lamella and bone. Tissue thickness was determined via digital micrometry (Mitutoyo 293-521; Mitutoyo, Tokyo, Japan). Then, the cartilage-only samples underwent unconfined compression tests using a mechanical testing machine (Zwick/Roell Z2.5; Zwick/Roell, Ulm, Germany) that was equipped with a compressive piston (20 mm diameter) and a load cell (200 N force range). The loading protocol employed a strain rate of 0.15% strain/s to a maximum strain of 21% [28] and assessed primarily the contribution of fluid pressurization and—to a lesser extent—the contribution of fibril reinforcement [29]. Displacement–load data were obtained (TestXpert, Zwick/Roell) and used to compute the Instantaneous Young Modulus (IYM) as the ratio of stress and strain by fitting a tangent to the strain range of 10–20%. Samples were kept hydrated throughout the measurements.

### 2.7. Statistical Analysis 

Statistical analysis was carried out by OS using SPSS Statistics (v28, Armonk, NY, US). δ_0_ denotes the respective parameter value in the unloaded configuration, while δ_var1_, δ_var2_, δ_var3_, δ_vlg1_, δ_vlg2_, and δ_vlg3_ refer to the parameter values in the various configurations of loading. Relative changes were determined by referencing the parameter values to δ_0_. Assuming normal distributions, measurements of ThC, meniscus extrusion, joint extension, deviation, and rotation were compared between each configuration using repeated-measures ANOVA followed by Tukey’s post-hoc test. Not assuming normal distributions, the absolute T2 and T1ρ values were compared using Friedman’s test followed by Dunn’s post-hoc test. To reduce the number of comparisons and to counteract the problem of multiple comparisons, post-hoc tests were performed only between the loaded compartment’s configurations, for example, between δ_0_, δ_var1_, δ_var2_, and δ_var3_ (in case of varus loading of the medial compartment), and the Bonferroni correction was applied. Additionally, the level of significance was set to *p* ≤ 0.01 to reduce the number of statistically significant, yet scientifically (most likely) irrelevant findings. 

## 3. Results

All knee joints underwent full MRI and CT imaging under loading and subsequent biomechanical and histologic referencing.

Mean ThC values decreased significantly in response to varus loading, both in the femoral and tibial cartilage of the MFTC (*p* < 0.001). Corresponding decreases were noted for the LFTC in response to valgus loading, even though statistical significance was only found for the femoral (*p* < 0.001) and not for the tibial cartilage (*p* = 0.117) (Table 2).

By and large, ThC gradually decreased with increasing loading intensity. Analysis of the distinct subregions corroborated these findings and indicated that significant decreases in ThC occurred primarily in the external, central, and internal subregions of the femoral and tibial cartilage (*p* ≤ 0.002) (Table A1 (Appendix A)).

In cartilage, T1ρ decreased under loading in both compartments (Table 3). Significant decreases in T1ρ were found for the lateral tibia only (*p* = 0.001), while for the medial femur, they tended towards significance (*p* = 0.034). These findings were confirmed by the subregional decreases in T1ρ that were significant in the internal, central, and peripheral regions of the lateral tibia (0.001 ≤ *p* ≤ 0.009) (Table A2 (Appendix A)).

In cartilage, T2 increased under loading in both compartments. These increases were statistically significant for the medial femur (*p* = 0.004) and the lateral tibia (*p* < 0.001) (Table 4). Subregionally, these increases were significant primarily in the central subregion (*p* ≤ 0.006) (Table A3 (Appendix A)).

Interestingly, loading-induced changes in ThC, T1ρ, and T2 tended to plateau between the δ_var2_ (or δ_vlg2_) and δ_var3_ (or δ_vlg3_) configurations despite increasing loading intensities. Consequently, no linear association of the respective parameters’ changes and increased loading intensities were found.

For the medial and lateral menisci (Table 5), loading-induced decreases in T1ρ were gradual and closely related to loading intensity. These decreases were significant for the medial meniscus under varus loading (*p* = 0.001) and tended towards significance for the lateral meniscus under valgus loading (*p* = 0.020). Loading-induced increases in T2 were not significant, neither for the medial (*p* = 0.247) nor for the lateral meniscus (*p* = 0.022). Similarly, meniscal extrusion increased slightly, yet not significantly under loading.

**Figure 2:**
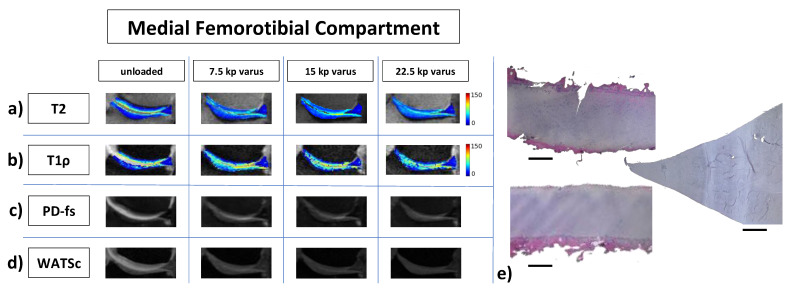
Representative multiparametric MR images of the medial femorotibial compartment in response to varus loading and corresponding histologic sections. (**a**–**d**) Displayed are T2 maps (**a**), T1ρ maps (**b**), and morphologic images, i.e., proton density-weighted fat-saturated (PD-fs, (**c**)) and water selective cartilage scan images (WATSc, (**d**)) as a function of increasing varus loading intensity. Segmentation outlines were overlaid onto the corresponding morphologic images and pixel intensities were color-coded (ms). MR images were cropped and zoomed to the areas of interest. (**e**) Corresponding histologic sections of the tibial cartilage (**e_1_**), the femoral cartilage (**e_2_**), and medial meniscus (**e_3_**) after hematoxylin-eosin staining. Bars indicate 1 mm. Same right knee joint as in Figure 1 and Figure 3.

**Figure 3:**
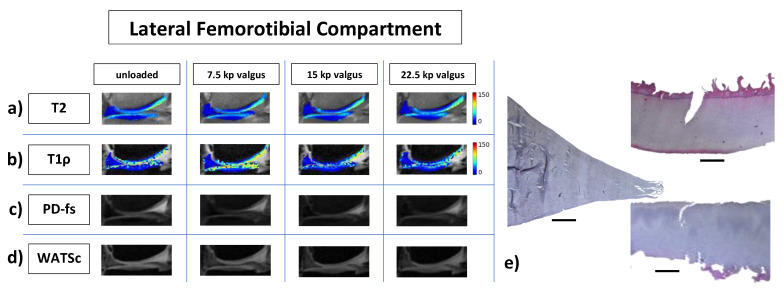
Representative multiparametric MR images of the lateral femorotibial compartment in response to varus loading and corresponding histologic sections. MR images (**a**–**d**) are organized as in Figure 2. For the histologic sections (**e**), the lateral meniscus (**e_1_**) is positioned between the femoral (**e_2_**) and tibial cartilage (**e_3_**). Same right knee joint as in Figure 1 and Figure 2.

Qualitatively, Figure 2 and Figure 3 indicate the loading-induced changes of the MFTC (Figure 2) and the LFTC (Figure 3) in a representative knee joint and in reference to respective histologic sections.

Joint alignment as assessed by CT was related to loading intensity and configuration (Table 6). As expected, joint deviation angles that were measured on the lateral side of the joint increased (or decreased) significantly under varus (or valgus) loading (*p* < 0.001), thereby indicating more varus and valgus morphotypes under loading. Joint extension angles decreased significantly under loading (*p* < 0.001), indicating steadily increasing joint flexion under loading, yet changes were more pronounced under varus than valgus loading. Joint rotation was significantly different, too, with external rotation observed under varus loading and internal rotation under valgus loading. Taken together, the joints tended to undergo moderate flexion and external rotation under varus loading as well as slight flexion and internal rotation under valgus loading.

Reference evaluation indicated variable, yet largely mild-to-moderate histologic degeneration of cartilage and meniscus and relatively uniform biomechanical properties of cartilage. Mean modified OARSI scores for cartilage were 1.9 ± 1.2 (range, 0–3) for the medial tibia, 2.1 ± 1.6 (range, 0–4.5) for the medial femur, 1.4 ± 1.3 (range, 0–3.5) for the lateral tibia, and 1.8 ± 1.2 (range, 0–4) for the lateral femur. These findings were reflected by mean Pauli grades for the medial (2.1 ± 1.1 (range, 1–4)) and lateral meniscus (2.3 ± 1.2 (range, 1–4)), indicating mild-to-moderate degeneration of both menisci. Mean IYM values of the cartilage-only samples were 1.0 ± 0.7 MPa (range, 0.3–2.1) for the medial tibia, 1.4 ± 1.0 MPa (range, 0.1–3.4) for the medial femur, 1.0 ± 1.1 MPa (range, 0.1–2.5) for the lateral tibia, and 1.0 ± 0.7 MPa (range, 0.3–2.4) for the lateral femur.

Because of the small sample size, correlations between image-based measures of tissue functionality, i.e., loading-induced changes in T2 or T1ρ, and histologic or biomechanical reference measures were not determined.

## 4. Discussion

The most important finding of this study is that, in situ, T1ρ and T2 are reflective of loading-induced adaptive changes of cartilage and meniscus under varus and valgus loading and thereby complement the morphologic and morphometric evaluation of these tissues. Moreover, this study defines normative values of the response-to-loading patterns in cartilage and meniscus as a function of the joint compartment, region, subregion and in reference to histologic and biomechanical measures.

Across the joint, T1ρ decreased in cartilage and meniscus under loading, even though these changes were only significant in the lateral tibia and medial meniscus. Considering the still unclear association of T1ρ with the distinct structural and compositional cartilage constituents, these changes are most likely brought about by a variety of processes. Some authors highlighted the association of T1ρ to tissue hydration [30], proteoglycan content [31], and collagen network properties [32] and the loading-induced changes in these tissue properties are likely responsible for the underlying changes in T1ρ. Literature data on changes of T1ρ under loading are heterogeneous, with some authors reporting decreases [33,34] and increases [35]. Nonetheless, decreases in T1ρ are plausible as cartilage is compressed under loading and its solid phase, largely made up of proteoglycan and collagen [36], is densified [37], thereby increasing the relative amount and density of proteoglycans and collagens and decreasing T1ρ values [38]. Additionally, rheological properties of the fluid phase, made up of the interstitial fluid [36], within this densified framework are consequently reduced [39] and fluid is secondarily redistributed within the tissue [40] as well as partially lost into the synovial fluid [37], which also decreases T1ρ values [41].

For T2, our findings were more ambiguous with variable and—in parts—undulating increases in articular cartilage and meniscus found in response to loading. These increases were only significant for the medial femur and lateral tibia, however. The exact correlates of T2 remain controversial, too, with associations to water content [42], concentration of proteoglycans [43] and collagens [44], and anisotropy of the collagen network [45] discussed in the literature. Similarly, the changes in cartilage and meniscus to be expected under loading remain controversial, too, with some authors reporting increases [6,46] and others reporting decreases [33,47]. Despite our best efforts, our study does not clarify this situation and indicates the need for additional research.

Likely, the substantial standard deviations prevented clearer inferences for both T2 and T1ρ. Yet, the overall changes under loading and, thus, the dynamic range were larger for T1ρ, which is in line with earlier reports [13]. This, in turn, confirms that the distinctly different structural, compositional, and biophysical profiles of both parameters translate to distinctly different mechanosensitivities.

Caution should be exercised when directly comparing our and other studies because differences in study design, experimental setup, and the imaging framework need to be considered. Most previous studies were conducted in vitro on excised cartilage samples [46,48,49] and did thus not consider the actual physiological interactions of cartilage and meniscus with the surrounding joint structures. Differences in MRI acquisition parameters, e.g., the number and spacing of echo times (for T2) and the number, duration, and amplitude of spin-lock pulses (for T1ρ), in-plane resolution and slice thickness, let alone variable post-processing methodologies, add another layer of complexity to the interpretation of these findings. While most previous studies focused on axial compression of the joint along the mechanical axis, we used varus and valgus loading perpendicular to the mechanical axis. Obviously, this type of loading is inherently different and characterized by substantially lower forces within the joint [15], thereby decreasing comparability, too. Another important aspect pertains to the coil to be used for imaging. In this study, standard two-element general-purpose coils as provided by the MRI vendor were used. When bearing the future in vivo application in mind, flexible coils provide a close fit around the joint and, thus, increased signal-to-noise ratio and patient comfort, which is a promising alternative for stress MRI of the knee joint [50,51].

Surprisingly, the quantitative values of ThC, T1ρ, and T2 were not linearly associated with loading intensity but plateaued for moderate and strong loading. Even though earlier studies investigating stress radiography of the knee joint applied various pressure levels ranging between 3 and 30 kp [52], our findings indicate that moderate loading of up to 15 kp is sufficiently efficient to study cartilage functionality, thereby corroborating earlier findings [15]. Higher loading intensities seem not justified in the context of functional joint imaging as the additional diagnostic benefit is unclear, while the risk of harm to the patient is increased.

Another important aspect to consider is the reversibility of the loading-induced changes in cartilage. During post-loading recovery, (ultra)structural and compositional recovery is guided by elastic and osmotic adaptive processes that eventually restore the tissue to its original shape and height, which involves equilibration and homoeostasis of associated biophysical processes. The time demand of the post-loading recovery is related to the loading magnitude, type, and other biomechanical parameters, and literature data indicate that it may be slow and that it may take minutes to hours until full recovery of the pre-loading configuration has been reached [34,49,53,54,55]. As the cartilage changes over time were not addressed in this study, future studies need to further assess the temporal interplay of mechanical loading and image acquisition in the interest of equally safe, efficient, and diagnostically beneficial clinical translation.

Biomechanical reference measures were relatively homogeneous which is most likely due to the fact that biomechanical properties of cartilage are more closely associated with extracellular matrix integrity than with extracellular matrix composition [56]. As cartilage was found to display mild-to-moderate degeneration, biomechanical properties were not expected to be substantially altered.

Our study had several limitations. First, this study’s in situ design limits transferability to the clinical setting as we only studied the joint’s static stabilizers, while not considering the joint’s dynamic stabilizers. Nonetheless, our findings may provide guidance and direction for future in vivo studies on volunteers and patients. Second, cartilage zones were not considered in this pilot study that aimed to assess the diagnostic value of T1ρ and T2 mapping by stress MRI, while keeping the amount of data manageable. Third, this study focused on T1ρ and T2 that are considered to be the most promising cartilage imaging techniques [3,57], yet did not consider other advanced MRI techniques such as delayed gadolinium-enhanced MRI of cartilage (dGEMRIC) [58], glycosaminoglycan Chemical Exchange Saturation Transfer (gagCEST) [59] or sodium imaging [60]. Fourth, we only included knee joint specimens of elderly body donors, which account for the tissues’ mild-to-moderate degeneration (as assessed histologically) and may be responsible for slightly different biomechanical behavior as compared to clinical populations [6]. Fifth, the joints underwent substantial adaptive motion and altered joint alignment that was related to the type and intensity of loading. Since the femur and tibia were not mechanically confined when loaded (unlike the in vivo situation in patients), the adaptive motion certainly changed the femorotibial contact areas that gradually moved posteriorly with increasing flexion, peripherally with increasing deviation, and variably with increasing rotation. Consequently, despite our best efforts to exactly match the mid-coronal imaging plane (for T2 and T1ρ quantification) with the histologic and biomechanical reference modalities, matching may have been inaccurate due to the loading-induced adaptive motion.

## 5. Conclusions

In conclusion, precise varus and valgus loading of the knee induces compartmental pressurization and alters the structure and composition of cartilage and meniscus that may be assessed by cartilage morphometry and T2 and T1ρ mapping. Once validated in future clinical and basic research studies, the response-to-loading patterns may be used as imaging surrogates of tissue functionality.

## Figures and Tables

**Table 1:** Acquisition parameters of the MRI sequences.

Sequence Parameters	PD-fs	WATSc	T2	T1ρ
Orientation	cor, ax (sag) *	cor	mid-cor	cor ***
Type of fat saturation	SPAIR	water-selective excitation	*n*/a	*n*/a
Sequence type	2D Turbo-spin-echo	3D Gradient echo	2D Multi-spin-echo	3D Spin-lock multi-gradient echo
Repetition time (ms)	4776 (ax)–7125 (sag)	10	1400	5
Echo time (ms)	30	5	*n* × 7.4 (*n* = 1–8) **	3
Turbo spin-echo factor	13 (ax)–15 (sag)	1	15	64
Field of view (mm)	180 × 180	180 × 180	180 × 180	180 × 180
Acquisition matrix (pixels)	368 × 368	368 × 368	368 × 368	368 × 368
Reconstruction matrix (pixels)	720 × 720	720 × 720	720 × 720	720 × 720
Pixel size (mm/pixel)	0.25 × 0.25	0.25 × 0.25	0.25 × 0.25	0.25 × 0.25
Scan percentage (%)	100	100	100	100
Flip angle (°)	90	17	90	10
Number of signal averages (*n*)	1	1	1	1
Slices (*n*)	30 (sag)–33 (ax)	266	1	30
Slice Thickness/Gap (mm)	3.0/0.5	1.5/0.0	3.0/*n*/a	3.0/0.0
Spin-lock durations (ms)	*n*/a	*n*/a	*n*/a	0, 10, 20, 30, 40
Duration (min)	4 min 37 s (ax)–5 min 56 s (sag)	7 min 53 s	8 min 38 s	7 min 36 s ***

Abbreviations: Spectral attenuated inversion recovery (SPAIR), Proton Density (PD), Water selective cartilage scans (WATSc), fat-saturated (fs), not applicable (*n*/a), (mid-)coronal ((mid-)cor), axial (ax), sagittal (sag). * PD-fs sequences were acquired in all three orientations in the unloaded reference configuration and in the coronal and axial orientations under loading. ** Although echo times of up to 112 ms (*n* = 15) were sampled, only the first eight echoes were included in the T2 quantification because of insufficiently low signal-to-noise ratio at longer echo times. *** Only the mid-coronal slice was included in the analysis.

**Table 2:** Mean absolute thickness of the tibial and femoral articular cartilage regions as a function of loading. Unloaded (δ_0_) and loaded to 7.5 kp (δ_var1_, δ_vlg1_), 15 kp (δ_var2_, δ_vlg2_), and 22.5 kp (δ_var3_, δ_vlg3_). Mean ± standard deviations (mm) (mean percentage change versus δ_0_ (%)). Values are given for the loaded compartment only, i.e., the medial compartment under varus loading and the lateral compartment under valgus loading. Statistical analysis was performed using repeated measures ANOVA with statistically significant findings highlighted in **bold type**.

Compartment	Region	δ_0_	δ_var1_ or _vlg1_	δ_var2_ or _vlg2_	δ_var3_ or _vlg3_	*p*-Value
Medial Compartment	Tibia	1.59 ± 0.21	1.56 ± 0.19 (−2.1)	1.53 ± 0.19 (−3.5)	1.53 ± 0.20 (−3.8)	**<0.001**
	Femur	1.58 ± 0.34	1.56 ± 0.36 (−1.3)	1.55 ± 0.36 (−1.9)	1.54 ± 0.36 (−2.7)	**<0.001**
Lateral Compartment	Tibia	1.93 ± 0.36	1.92 ± 0.35 (−0.5)	1.91 ± 0.36 (−1.2)	1.89 ± 0.35 (−2.1)	0.117
	Femur	1.79 ± 0.32	1.78 ± 0.28 (−0.1)	1.75 ± 0.29 (−1.7)	1.77 ± 0.28 (−1.0)	**<0.001**

**Table 3:** Mean absolute T1ρ relaxation times of femoral and tibial articular cartilage in response to loading. Mean ± standard deviation (ms) (mean percentage change versus δ_0_ (%)). Statistical analysis was performed using Friedman’s test with statistically significant findings highlighted in **bold type**. Please refer to Table 2 for additional details on table organization.

Compartment	Region	δ_0_	δ_var1_ or _vlg1_	δ_var2_ or _vlg2_	δ_var3_ or _vlg3_	*p*-Value
Medial Compartment	Tibia	55.8 ± 33.7	42.7 ± 17.0 (−23.5)	39.0 ± 12.0 (−30.1)	36.9 ± 12.3 (−33.9)	0.675
	Femur	89.7 ± 30.7	60.6 ± 15.5 (−32.4)	62.8 ± 18.7 (−30.0)	60.8 ± 26.9 (−32.2)	0.034
Lateral Compartment	Tibia	60.6 ± 31.0	46.3 ± 18.6 (−23.6)	38.5 ± 18.6 (−36.5)	37.2 ± 17.4 (−38.6)	**0.001**
	Femur	73.4 ± 23.5	63.9 ± 18.4 (−12.9)	60.3 ± 19.1 (−17.8)	62.8 ± 20.4 (−14.4)	0.761

**Table 4:** Mean absolute T2 relaxation times of femoral and tibial articular cartilage in response to loading. Mean ± standard deviation (ms) (mean percentage change versus δ_0_ (%)). Statistical analysis was performed using Friedman’s test with statistically significant findings highlighted in **bold type**. Please refer to Table 2 for additional details on table organization.

Compartment	Region	δ_0_	δ_var1_ or _vlg1_	δ_var2_ or _vlg2_	δ_var3_ or _vlg3_	*p*-Value
Medial compartment	Tibia	30.9 ± 7.9	30.6 ± 9.2 (−1.0)	33.3 ± 13.2 (7.8)	33.1 ± 11.0 (7.1)	0.239
	Femur	36.4 ± 8.8	40.0 ± 11.6 (9.9)	41.5 ± 11.2 (14.0)	42.9 ± 12.0 (17.9)	**0.004**
Lateral Compartment	Tibia	29.6 ± 6.4	33.3 ± 7.5 (12.5)	30.1 ± 6.0 (1.7)	31.9 ± 9.7 (7.8)	**<0.001**
	Femur	38.6 ± 5.8	39.5 ± 7.3 (2.3)	39.5 ± 7.7 (2.3)	39.4 ± 6.4 (2.1)	0.366

**Table 5:** Mean absolute T1ρ and T2 relaxation times (ms) and meniscal extrusion (mm) of the medial and lateral meniscus body region in response to varus and valgus loading. Mean ± standard deviation (mean percentage change versus δ_0_ (%)). Statistical analysis was performed using Friedman’s test for T1ρ and T2 and repeated measures ANOVA for meniscal extrusion. Statistically significant results are indicated by **bold type**. Please refer to Table 2 for additional details on table organization.

Meniscus	Parameter	δ_0_	δ_var1_ or _vlg1_	δ_var2_ or _vlg2_	δ_var3_ or _vlg3_	*p*-Value
Medial	T1ρ (ms)	24.7 ± 6.2	21.6 ± 8.5 (−12.6)	20.1 ± 8.0 (−18.6)	17.5 ± 8.9 (−29.1)	**0.001**
	T2 (ms)	14.4 ± 2.1	15.8 ± 3.4 (9.7)	15.8 ± 3.6 (9.7)	16.3 ± 3.6 (13.2)	0.247
	Extrusion (mm)	3.3 ± 1.2	3.3 ± 1.1 (0)	3.3 ± 1.2 (0)	3.4 ± 1.4 (3.0)	0.325
Lateral	T1ρ (ms)	18.9 ± 4.8	17.4 ± 4.5 (−7.9)	15.8 ± 5.5 (−16.4)	15.7 ± 5.5 (−16.9)	0.020
	T2 (ms)	13.1 ± 3.1	15.6 ± 3.0 (19.1)	15.3 ± 3.9 (16.8)	14.6 ± 3.8 (11.5)	0.022
	Extrusion (mm)	1.6 ± 0.8	1.7 ± 1.3 (6.3)	1.8 ± 1.2 (12.5)	1.7 ± 1.2 (6.3)	0.793

**Table 6:** Mean values of joint alignment, i.e., joint deviation (in the coronal plane), joint extension (in the sagittal plane), and joint rotation (in the axial plane) as a function of loading. Mean ± standard deviation (°). Statistical analysis was performed using repeated measures ANOVA and statistically significant results are indicated in **bold type**.

Parameter	δ_0_	δ_var1_	δ_var2_	δ_var3_	δ_vlg1_	δ_vlg2_	δ_vlg3_	*p*-Value
Joint deviation (°)	178.5 ± 4.0	180.2 ± 4.8	183.1 ± 7.1	186.4 ± 7.6	174.9 ± 3.4	171.9 ± 2.5	167.1 ± 14.9	**<0.001**
Joint extension (°)	162.7 ± 10.6	159.8 ± 10.5	154.8 ± 11.7	150.9 ± 13.3	163.8 ± 8.1	162.1 ± 10.8	161.1 ± 11.6	**<0.001**
Joint rotation (°)	7.7 ± 5.6	11.3 ± 7.4	15.5 ± 10.1	21.7 ± 14.5	–1.5 ± 12.7	–2.2 ± 17.1	–3.7 ± 20.4	**<0.001**

**Figure A2:**
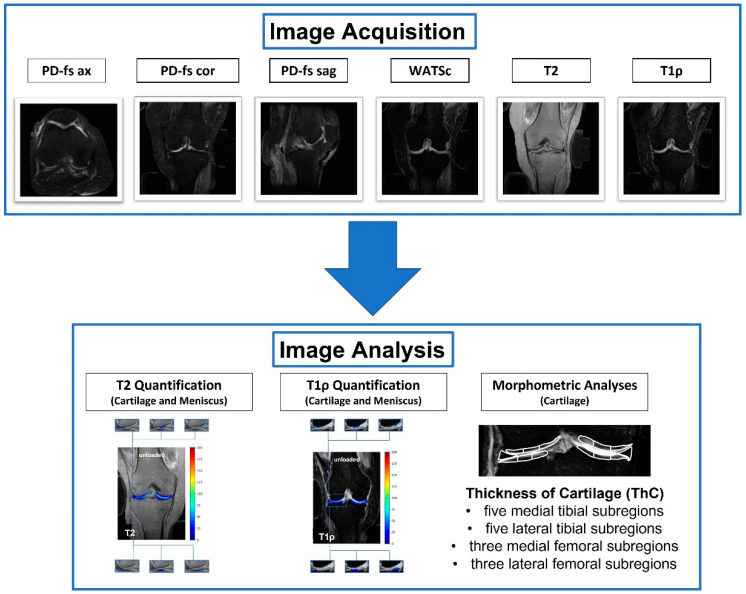
Workflow of image acquisition and analysis. For each joint specimen and loading configuration, PD-weighted fat-saturated (PD-fs) sequences were acquired in the axial (ax), coronal (cor) and sagittal (sag) orientation as well as 3D water-selective cartilage scans (WATSc), and quantitative T2 and T1ρ mapping sequences. The MR data were then analysed to reconstruct quantitative T2 and T1ρ maps, while morphologic sequences were used for segmentation and morphometric analyses.

## Data Availability

The data not contained in the manuscript or appendix is available from the corresponding author upon reasonable request.

## Appendix A

**Table A1:** Mean cartilage thickness of the distinct subregions of the medial and lateral femur and tibia as a function of loading. Mean ± standard deviation (mm) (mean percentage change versus δ_0_ (%)). Statistical analysis was performed using repeated measures ANOVA and statistically significant findings are highlighted in **bold type**. Abbreviations: central medial femur (cMF), central lateral femur (cLF), medial tibia (MT), lateral tibia (LT). Please refer to Table 2 for additional details on table organization.

Compartment	Region	Subregion	δ_0_	δ_var1_ or _vlg1_	δ_var2_ or _vlg2_	δ_var3_ or _vlg3_	*p*-Value
Medial Compartment	Tibia	Central subregion of MT	2.14 ± 0.39	2.10 ± 0.37 (−1.9)	2.06 ± 0.35 (−3.6)	2.04 ± 0.35 (−4.5)	**0.002**
		External subregion of MT	1.25 ± 0.26	1.21 ± 0.29 (−3.2)	1.18 ± 0.29 (−5.8)	1.20 ± 0.31 (−4.6)	**<0.001**
		Internal subregion of MT	1.79 ± 0.22	1.76 ± 0.20 (−1.7)	1.73 ± 0.20 (−3.4)	1.73 ± 0.20 (−3.4)	**<0.001**
		Anterior subregion of MT	1.54 ± 0.19	1.51 ± 0.18 (−1.9)	1.52 ± 0.19 (−1.4)	1.50 ± 0.20 (−2.6)	0.124
		Posterior subregion of MT	1.30 ± 0.22	1.27 ± 0.19 (−2.6)	1.25 ± 0.20 (−4.2)	1.26 ± 0.21 (−3.4)	0.104
	Femur	Central subregion of cMF	1.93 ± 0.40	1.90 ± 0.44 (−1.3)	1.89 ± 0.44 (−2.0)	1.87 ± 0.46 (−2.8)	**0.002**
		External subregion of cMF	1.20 ± 0.29	1.17 ± 0.30 (−2.4)	1.17 ± 0.30 (−2.8)	1.14 ± 0.30 (−5.3)	**<0.001**
		Internal subregion of cMF	1.62 ± 0.45	1.62 ± 0.46 (−0.1)	1.60 ± 0.45 (−1.5)	1.61 ± 0.45 (−1.0)	0.011
Lateral Compartment	Tibia	Central subregion of LT	3.06 ± 0.71	3.02 ± 0.64 (−1.2)	3.00 ± 0.67 (−1.7)	2.97 ± 0.64 (−2.9)	0.256
		External subregion of LT	1.44 ± 0.20	1.44 ± 0.19 (−0.1)	1.40 ± 0.21 (−2.8)	1.40 ± 0.21 (−2.8)	0.303
		Internal subregion of LT	1.91 ± 0.36	1.89 ± 0.39 (−1.1)	1.90 ± 0.39 (−0.3)	1.89 ± 0.38 (−1.3)	0.426
		Anterior subregion of LT	1.66 ± 0.28	1.65 ± 0.26 (−1.0)	1.64 ± 0.27 (−1.6)	1.63 ± 0.29 (−2.2)	0.141
		Posterior subregion of LT	1.64 ± 0.39	1.67 ± 0.38 (1.9)	1.65 ± 0.39 (0.4)	1.61 ± 0.36 (−2.0)	0.103
	Femur	Central subregion of cLF	2.19 ± 0.44	2.19 ± 0.36 (0.1)	2.16 ± 0.36 (−1.6)	2.18 ± 0.36 (−0.7)	**<0.001**
		External subregion of cLF	1.52 ± 0.28	1.50 ± 0.28 (−1.1)	1.46 ± 0.27 (−3.7)	1.47 ± 0.26 (−3.1)	**<0.001**
		Internal subregion of cLF	1.67 ± 0.34	1.68 ± 0.32 (0.5)	1.67 ± 0.33 (−0.2)	1.68 ± 0.31 (0.6)	0.288

**Table A2:** Mean absolute T1ρ relaxation times of femoral and tibial cartilage subregions in response to loading. Mean ± standard deviation (ms) (mean percentage change versus δ_0_ (%)). Statistical analysis was performed using Friedman’s test and statistically significant findings are highlighted in **bold type**. Please refer to Table 2 for additional details on table organization.

Compartment	Region	Subregion	δ_0_	δ_var1_ or _vlg1_	δ_var2_ or _vlg2_	δ_var3_ or _vlg3_	*p*-Value
Medial compartment	Tibia	Internal third of MT	43.8 ± 22.4	35.6 ± 18.0 (−18.7)	33.4 ± 10.6 (−23.7)	31.6 ± 8.8 (−27.9)	0.092
		Central third of MT	56.7 ± 36.4	46.8 ± 22.3 (−17.5)	41.8 ± 18.3 (−26.3)	36.8 ± 17.0 (−35.1)	0.772
		Peripheral third of MT	71.5 ± 46.6	51.2 ± 21.1 (−28.4)	44.7 ± 14.3 (−37.5)	46.5 ± 26.6 (−35.0)	0.754
	Femur	Internal third of MF	97.5 ± 28.3	65.0 ± 24.4 (−33.3)	69.1 ± 24.6 (−29.1)	69.1 ± 35.8 (−29.1)	0.186
		Central third of MF	81.8 ± 32.3	61.6 ± 18.3 (−24.7)	61.7 ± 21.0 (−24.6)	57.4 ± 27.2 (−29.8)	0.138
		Peripheral third of MF	87.7 ± 42.3	50.4 ± 13.9 (−41.1)	49.9 ± 18.8 (−42.5)	49.5 ± 24.8 (−43.6)	0.064
Lateral compartment	Tibia	Internal third of LT	69.5 ± 34.7	49.4 ± 20.9 (−28.9)	43.9 ± 26.0 (−36.8)	36.4 ± 20.0 (−47.6)	**0.006**
		Central third of LT	50.4 ± 30.5	39.9 ± 17.1 (−20.8)	29.7 ± 13.8 (−41.1)	34.7 ± 17.1 (−31.2)	**0.001**
		Peripheral third of LT	62.2 ± 30.6	50.9 ± 25.1 (−18.2)	44.9 ± 26.2 (−27.8)	40.9 ± 22.8 (−34.2)	**0.009**
	Femur	Internal third of LF	78.6 ± 31.8	73.4 ± 25.8 (−6.6)	78.8 ± 33.1 (0.3)	80.9 ± 27.9 (2.9)	0.768
		Central third of LF	70.2 ± 27.0	60.1 ± 17.6 (−14.4)	51.1 ± 14.3 (−27.2)	53.4 ± 16.5 (−23.9)	0.674
		Peripheral third of LF	67.9 ± 17.4	54.3 ± 20.9 (−20.0)	45.5 ± 18.8 (−33.0)	47.4 ± 22.9 (−30.2)	0.053

**Table A3:** Mean absolute T2 relaxation times of femoral and tibial cartilage subregions in response to loading. Mean ± standard deviation (ms) (mean percentage change versus δ_0_ (%)). Statistical analysis was performed using Friedman’s test and statistically significant findings are highlighted in **bold type**. Please refer to Table 2 for additional details on table organization.

Compartment	Region	Subregion	δ_0_	δ_var1_ or _vlg1_	δ_var2_ or _vlg2_	δ_var3_ or _vlg3_	*p*-Value
Medial compartment	Tibia	Internal third of MT	25.9 ± 7.5	24.8 ± 8.8 (−4.2)	28.7 ± 12.3 (10.8)	29.7 ± 10.9 (14.7)	0.011
		Central third of MT	31.1 ± 10.4	30.4 ± 9.9 (−2.3)	34.3 ± 17.9 (10.3)	32.8 ± 14.4 (5.5)	0.990
		Peripheral third of MT	38.7 ± 7.8	40.4 ± 9.1 (4.4)	39.5 ± 9.3 (2.1)	39.4 ± 7.8 (1.8)	0.081
	Femur	Internal third of MF	39.9 ± 11.1	44.1 ± 10.7 (10.5)	44.7 ± 10.9 (12.0)	46.4 ± 10.1 (16.3)	0.186
		Central third of MF	32.7 ± 8.0	36.4 ± 14.5 (11.3)	38.4 ± 13.7 (17.4)	40.1 ± 14.8 (22.6)	**0.005**
		Peripheral third of MF	35.4 ± 8.1	37.5 ± 10.0 (5.9)	39.9 ± 10.6 (12.7)	40.8 ± 16.6 (15.3)	0.013
Lateral compartment	Tibia	Internal third of LT	31.6 ± 9.2	38.1 ± 11.4 (20.6)	31.8 ± 9.5 (0.6)	35.4 ± 11.6 (12.0)	**0.006**
		Central third of LT	24.8 ± 4.4	25.1 ± 5.9 (1.2)	24.1 ± 4.4 (−2.8)	24.3 ± 8.2 (−2.0)	**<0.001**
		Peripheral third of LT	36.9 ± 10.2	40.7 ± 9.3 (10.3)	38.9 ± 7.6 (5.4)	39.5 ± 11.1 (7.0)	0.105
	Femur	Internal third of LF	44.2 ± 9.4	44.7 ± 9.8 (1.1)	44.6 ± 12.8 (0.9)	44.9 ± 11.3 (1.6)	0.782
		Central third of LF	35.9 ± 8.2	36.3 ± 8.0 (1.1)	35.3 ± 6.9 (−1.7)	35.0 ± 6.4 (−2.5)	0.131
		Peripheral third of LF	34.2 ± 4.6	36.3 ± 6.3 (6.1)	37.1 ± 5.8 (8.5)	38.2 ± 3.9 (11.7)	0.019
